# Spatial energy density of large-scale electricity generation from power sources worldwide

**DOI:** 10.1038/s41598-022-25341-9

**Published:** 2022-12-08

**Authors:** Jonas Kristiansen Nøland, Juliette Auxepaules, Antoine Rousset, Benjamin Perney, Guillaume Falletti

**Affiliations:** 1 Department of Electric Power Engineering (IEL), Norwegian University of Science and Technology (NTNU), O. S. Bragstads plass 2E, 7034 Trondheim, Norway; 2 Grenoble Institute of Technology (Grenoble INP), Grenoble, 38031 France

**Keywords:** Electrical and electronic engineering, Energy infrastructure

## Abstract

This paper introduces the annual energy density concept for electric power generation, which is proposed as an informative metric to capture the impacts on the environmental footprint. Our investigation covers a wide range of sources classified by rated power and compares different regions to establish typical spatial flows of energy and evaluate the corresponding scalability to meet future net-zero emission (NZE) goals. Our analysis is conducted based on publicly available information pertaining to different regions and remote satellite image data. The results of our systematic analysis indicate that the spatial extent of electric power generation toward 2050 will increase approximately sixfold, from approximately 0.5% to nearly 3.0% of the world’s land area, based on International Energy Agency (IEA) NZE 2050 targets. We investigate the worldwide energy density for ten types of power generation facilities, two involving nonrenewable sources (i.e., nuclear power and natural gas) and eight involving renewable sources (i.e., hydropower, concentrated solar power (CSP), solar photovoltaic (PV) power, onshore wind power, geothermal power, offshore wind power, tidal power, and wave power). In total, our study covers 870 electric power plants worldwide, where not only the energy density but also the resulting land or sea area requirements to power the world are estimated. Based on the provided meta-analysis results, this paper challenges the common notion that solar power is the most energy-dense renewable fuel source by demonstrating that hydropower supersedes solar power in terms of land use in certain regions of the world, depending on the topography.

## Introduction

Due the to rising energy needs and changing energy mix, the spatial extent of the area required for electricity generation has recently received increasing attention^1,2^. In 2015, Smil^1^ provided quantitative estimates in terms of the order of magnitude of the mean power density of renewable flows, which captures the spatial concentration of power. These estimates are summarized in Table 1 and recalculated considering the concept of the annual energy density, which is introduced in this paper as a metric explaining the annual electric energy that can be generated for a given amount of regulated site area of the power plant.

van Zalk and Behrens^3^ quantitatively improved Smil’s^1^ mean power density estimates and performed a meta-analysis of 54 eligible studies of nonrenewable and renewable power generation facilities. However, the data provided were exclusively limited to the US. This paper goes much further by establishing a comprehensive population study of 870 power generation facilities worldwide to reveal significant geographical variations and includes other renewable sources, such as wave, tidal, and hybrid renewable offshore parks, to utilize natural marine resources^4^. We challenge the current consensus that solar energy exhibits the highest power density among renewable technologies^5^. It is revealed that there exist significant differences between different regions, depending on the topography and solar irradiation intensity^6^, impacting the affordability^7^. A detailed meta-analysis of electric power generation from bioenergy is beyond the scope of this paper. A thorough population study of the biomass energy density for electric power generation ($n = 63$) has already been provided^3^. Even though bioenergy constitutes a dilute resource, it could be utilized involving surplus biomass originating from other production processes, thus reducing environmental impacts^8,9^.

**Table 1:** Typical order of magnitude of the specific power of renewable flows^1^ and recalculated into the annual energy density.

Metric	Solar heat	Solar PV	Hydro	Wind	Biomass
Mean power ($p_g$)	$\sim 10^2$ W/m$^{2}$	$\sim 10^1$ W/m$^{2}$	$\sim 10^0$ W/m$^{2}$	$\sim 10^{-1}$ W/m$^{2}$	$\sim 10^{-1}$ W/m$^{2}$
Ann. energy ($\varepsilon _g$)	$\sim 10^0$ TWh/km$^{2}$	$\sim 10^{-1}$ TWh/km$^{2}$	$\sim 10^{-2}$ TWh/km$^{2}$	$\sim 10^{-3}$ TWh/km$^{2}$	$\sim 10^{-3}$ TWh/km$^{2}$

Spatial power density evaluation is a topic of relevance to the field of life cycle assessment (LCA). In power generation LCA, not only is the power plant itself considered but also the land used for the mining of energy fuel sources, minerals, construction materials, waste handling, and plant decommissioning^10^. This process considers the fuel cycle and land use intensity throughout the entire lifetime of a facility, including direct and indirect impacts^11^. Earlier, this method was applied in the entire US to estimate the footprint of different sources needed to produce a given amount of energy^12^. Moreover, a more recent detailed-level analysis of natural gas land use types in Texas has been published^13^. In general, energy sprawl has been found to be the key driver of land use increase for energy development in the US^14^. Other environmental concerns included pollution-related impacts such as greenhouse gas emissions, freshwater ecotoxicity, eutrophication, and particulate-matter exposure, in which renewable energies were particularly beneficial^15^. In present LCA studies, the plant-side power density of different types of facilities worldwide has not been closely studied.

In general, it is challenging to develop a common definition of the energy density and the spatial extent of different power sources. These quantities also vary over time, e.g., the power density of photovoltaic (PV) generation decreases one order of magnitude from noon to the annual average level. Therefore, an average power density is often defined, while this paper proposes the annual energy density independent of temporal variations. Our work also presents a transparent framework for the definition of the land requirements of power generation. For example, the land requirements of hydropower generation can vary to a high degree, depending on whether one considers the catchment area or only the footprint of the reservoir. Similarly, nuclear power generation must include the exclusion zone, and natural gas power plants must consider the land occupation of pipelines and mining.

Power generation facilities exert a myriad of other important environmental impacts on the local environment^16^ that are not considered herein. The implications of our investigation of the annual energy density include not only the resulting land requirements but also the spatial extent of the power infrastructure needed to generate energy. Another factor is that the land use of different power sources tends to be perceived differently^17^, as hydropower and solar power are associated with a significantly lower negative perception than that associated with wind power. Solar power can be utilized in combination with agricultural land to potentially maximize the benefits of a given land use^18^. Moreover, distributed solar power generation on residential rooftops utilizes existing available surfaces to harvest energy^19^. Even though building-integrated solar power generation to a certain extent can solve the problem of land use by utilizing existing surfaces^19,20^, solar power is generated at the low-voltage end of the power grid but can still help reduce local energy needs. Nevertheless, this technology is not perceived as a large-scale power generation solution due to its distributed nature. However, this approach can reduce the future demand for electricity in residential applications, which can liberate capacity from large-scale generation.

**Table 2:** Calculated shares of the world energy use^21^ and electric power generation^22^ in 2019 and 2020.

Sector	Year	Wave (%)	Tidal (%)	Hydro (%)	Wind (%)	Solar (%)	Bio (%)	Geo (%)	Nuclear (%)	Coal (%)	Oil (%)	Gas (%)
Energy use	2019	0.0	0.0	6.5	2.2	1.1	1.5	0.1	4.3	27.1	33.0	24.2
	2020	0.0	0.0	6.9	2.5	1.4	1.6	0.1	4.3	27.2	31.3	24.7
Electric gen.	2019	0.0	0.0	15.9	5.3	2.6	2.5	0.3	10.4	36.5	3.0	23.5
	2020	0.0	0.0	16.5	5.9	3.2	2.7	0.4	10.1	35.2	2.8	23.2

There are also known ways to significantly improve the power density of renewable sources^23^. However, this paper focuses on real-world power generation facilities (or at least considers prototypes in the case of wave and tidal power generation), suggesting that low-technology readiness level (TRL) technologies are beyond the research scope. Another example of facilities not considered in this paper is run-of-river hydropower plants, which occupy less land. However, the power capabilities of these hydropower plants may be simultaneously restricted in all regions to the minimum stream flow, which challenges the supply security. Large water reservoirs inherently occupy more space but override this drawback by providing firm dispatchable power to the electricity grid and supporting the energy security. As indicated in Table 2, the relative share of fossil fuels in primary energy and electricity use remains very high and must be significantly shifted to meet net-zero emission (NZE) goals toward 2050^22^. The impacts on the US alone have been presented in a 2021 Princeton University report indicating a significant increase in land requirements needed to achieve a zero-carbon US economy^24^. This paper focuses on the worldwide energy transition, thereby analyzing and projecting the energy density and spatial extent of the global electric power generation fleet.

The present paper is organized as follows: first, the mathematical relationships used for the calculations are described in the “Methods” section. Then, an analysis of the two above nonrenewable sources is presented. The above eight renewable sources are evaluated in the following section. Power and energy densities are summarized and compared in terms of their implications for land and sea requirements, and finally, conclusions are outlined.

## Methods

This section presents the data collection process and provides the mathematical relationships needed to perform the calculations and analyze the data presented in this paper.

### Data collection

**Figure 1:**
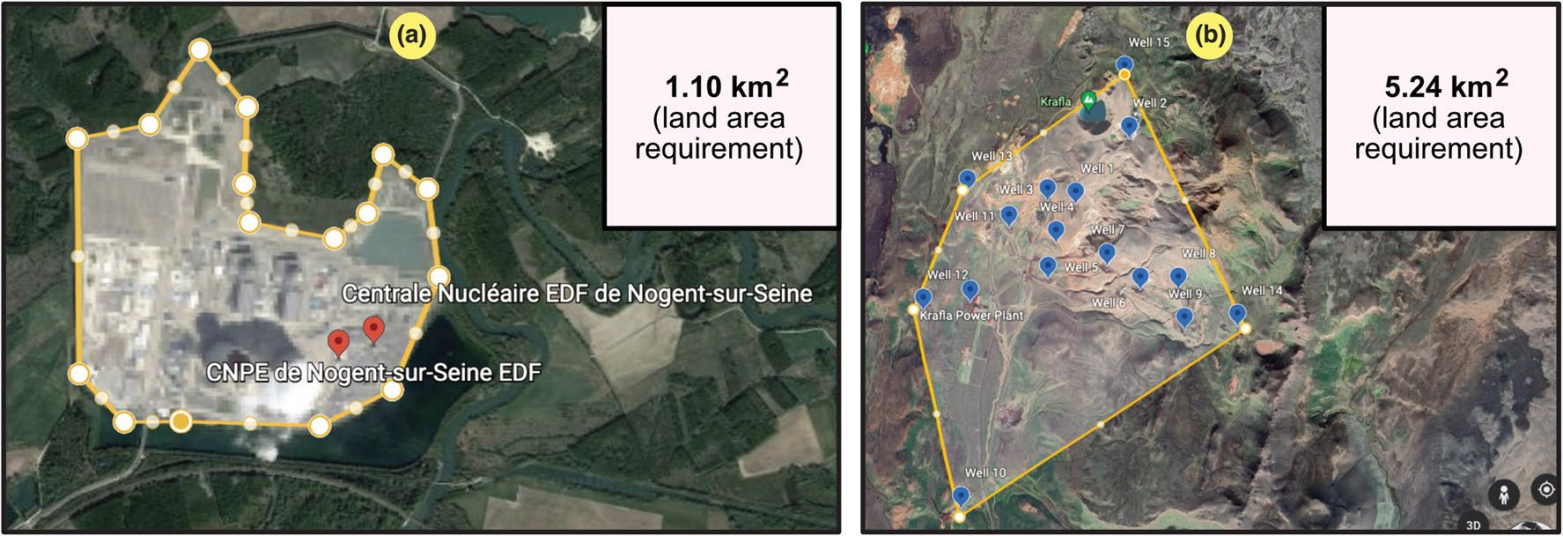
Example cases of the use of the Google Earth Engine^25^, version 7.3.2.5776 (https://earth.google.com/web/), to estimate the land area requirement for power generation facilities. (**a**) Nuclear power plant (in Nogent-sur-Seine, France). (**b**) Geothermal power plant (in Krafla, Iceland).

The process to obtain data was as follows: the individual pieces of information gathered for each power generation facility included in this paper were retrieved from publicly available information (e.g., Global Energy Observatory, Wikipedia, power producer nameplate sheets, etc.), including the rated power ($P_g$) and energy production ($E_g$). The land use requirement ($A_g$) for each facility was estimated from satellite images using the Google Earth Engine for spatially obtained measurements customized from high-resolution satellite data^25^. Sporadically, the total regulated area was also published. As there are alternate ways to define land requirements, Table 3 provides an overview of the lower and upper estimates found in the literature. Our chosen definitions of the spatial extent of the different power sources are bold and will be described in detail in later sections in this paper. All collected data are depicted in scatter plots in this article, and the values are provided in Supplementary Information files S1, separated by each power source. Two examples of area estimation are depicted in Fig. 1, illustrating the land use requirements for nuclear and geothermal power plants.

**Table 3:** Summary of the definitions proposed to establish land and sea requirements for the different power sources and technologies with references to existing studies in the literature, providing a comparison between lower and upper estimates.

Source	Lower estimate	Upper estimate
Natural gas Nuclear Geothermal Hydro	Gas-fired power plant for generation only^1^ Atomic reactor and steam turbine only^1^ Wells and thermal power plant only^1^ **Reservoir surface area**^1^	**Incl. delivery infrastructure and fuel extraction**^3,26^ **Incl. exclusion area for safety (and fuel handling)**^3,26^ **Incl. intermediate area between wells**^3^ Water catchment area^27^
Solar (CSP) Solar (PV) Solar (roof)$^{\text {a}}$ Wind Tidal Wave	Space for mirrors and tower^26^ On-ground PV area only on dual-purpose land^28^ **Zero occupied area by using existing surfaces** Direct impact of towers and infrastructure only^29,30^ **Pool surface only**^33,34^ **Wave absorber and converter only**^33^	**Incl. supporting infrastructure and regulated area** **Incl. regulated intermediate area between panels**^26^ Land use for PV material mining only^26^ **Total regulated area incl. indirect impacts**^3,31,32^ Incl. dam, added infrastructure and grid connections Incl. supporting infrastructure and grid connections

The bold values indicate the land use definitions considered in this paper.

$^{\text {a}}$Even though the land use for rooftop solar PV is low via the utilization of existing surfaces, the energy density of the actual surface must still be used to evaluate the scalability of residential distributed generation.

### Mathematical framework

Equations (1)–(3) cover the capacity factor and mean power density of consumption and generation, and their approximate energy balancing.

1 $$\begin{aligned} C_g&= \frac{E_g}{P_g T_y}, \quad&C_c&= \frac{E_c}{P_c T_y}, \end{aligned}$$

2 $$\begin{aligned} p_g&= C_g \frac{P_g}{A_g}, \quad&p_c&= C_c \frac{P_c}{A_{tot}}, \end{aligned}$$

3 $$\begin{aligned} C_g P_g&\approx C_c P_c, \quad&E_g&\approx E_c. \end{aligned}$$

The energy densities of consumption and generation can be determined from their respective mean power densities according to Eq. (4). Both metrics can be used to obtain the land use fraction to generate the needed energy over the total land area, as formulated in Eq. (5). Both of these surface areas are defined in Eq. (6).

4 $$\begin{aligned} \varepsilon _g&= p_g T_y, \quad&\varepsilon _c&= p_c T_y, \end{aligned}$$

5 $$\begin{aligned} \frac{A_g}{A_{tot}}&\approx \frac{p_c}{p_g}, \quad&\frac{A_g}{A_{tot}}&\approx \frac{\varepsilon _c}{\varepsilon _g}, \end{aligned}$$

6 $$\begin{aligned} A_g&= \frac{E_g}{\varepsilon _g} = C_g \frac{P_g}{p_g}, \quad&A_{tot}&= \frac{E_c}{\varepsilon _c} = C_c \frac{P_c}{p_c}. \end{aligned}$$

When electricity is generated, the actual power flow is higher, depending on the efficiency, as formulated in Eq. (7). This indicates that the mean specific power is influenced not only by the capacity factor but also by the electric energy conversion efficiency.

7 $$\begin{aligned} P_g = \eta P_s, \quad p_g&= \eta _g C_g \frac{P_s}{A_{g}}. \end{aligned}$$

## Nonrenewable resources

This section covers the two nonrenewable sources considered for electricity generation in this paper. While nuclear power can contribute to the net-zero climate target, natural gas is not a carbon-free resource without carbon capture and storage (CCS).

### Natural gas

Natural gas is an energy source that usually comprises a mix of methane, ethane, and propane, with methane as the major component. Electricity is generated via thermal power plants, which account for approximately one-third of the total natural gas energy use. In 2010, approximately 80% of the world’s electricity was produced in thermal power generation facilities.

**Figure 2:**
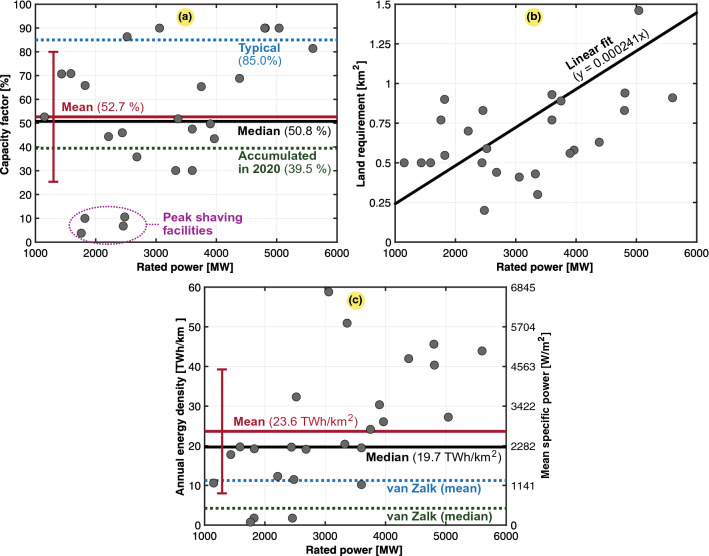
Scatter plots of 26 large- and medium-sized natural gas combined-cycle electric power plant facilities in the world (13 in the US, 5 in Japan, 4 in Russia, 2 in the United Arab Emirates, and 2 in Vietnam). Typical, mean, median, and fitted values are also included. The subplots are as follows: (**a**) capacity factor ($C_g$); (**b**) land use ($A_g$); and (**c**) annual energy density ($\varepsilon _g$) or mean specific power ($p_g$) excl. pipelines with van Zalk and Behrens^3^ as a reference.

A total of 26 natural gas electricity-generating power plants are shown in Fig. 2. Statistical analysis was performed to determine key data metrics. The case study includes the largest power plants worldwide in terms of the installed rated power. Figure 2a shows a scatter plot of the obtained capacity factors, and some factor values were very low, as these power plants were merely used for peak shaving, while the capacity factors for other power plants were closer to the typical value of 85%. The accumulated capacity factor of the worldwide natural gas fleet for 2020 was 39.5%, based on an overall rated power of 1830 GW and a total generation of 6333 TWh^35^. As shown in Fig. 2a, this value is slightly lower than the mean and median of the population we studied. As the capacity factor influences the power density, outliers could reduce the mean annual energy density of natural gas. A scatter plot of the land requirement was fitted against the rated power rating, as shown in Fig. 2b. Even though most of the points deviate from the linear fit, the trend is visible. They scale similarly in terms of their land requirement per power plant rating, and the land use consistently increased with the rated power. Finally, the scatter plot in Fig. 2c shows that the data exhibit a high standard deviation in terms of the power and energy density. Comparisons against van Zalk and Behrens^3^ are included, which is generally lower since their meta-analysis considered studies that took into account land use from the whole value chain of natural gas.

The overall results for the natural gas combined-cycle power plants are presented in Table 4. We observe that the overall power and energy density mean values of the entire population are relatively high (23.634 ${{\text {TWh/km}}^2}$ or 2696.17 ${\text{W/m}}^{2}$). It should be noted that these numbers consider only the power plant surface. When considering the rest of the fuel cycle, including natural gas production pads, underground fuel extraction is perceived not to entail high land requirements^10^. However, according to a recent study involving all the fuel cycle stages, the land requirement for production sites was approximately 1.44 times higher than that of the power plants themselves^13^. Table 5 provides the relative contribution to each of the stages, where several were determined insignificant, and thus, will be neglected in this paper. As indicated in Table 5, the highest contributor to land use is the delivery infrastructure of natural gas, which significantly reduces the final power and energy density values obtained. In this paper, we incorporated this effect by reviewing natural gas pipeline infrastructure estimates globally. The calculation steps needed are provided in Table 6, which starts with the worldwide energy use and electricity generation from natural gas. Then, the total pipeline length and its land requirement are obtained before the power and energy densities, including pipelines, are calculated. We could observe that the obtained end results were approximately seven to fifteen times lower than the previous mean value computed with only the surface of natural gas power plants. Notably, pipelines were not negligible, reflecting the implications of the initial assumptions we made, where the surface of pipelines was not considered. Considering that most of the natural gas pipelines worldwide are buried underground^36^, we hereafter use the upper estimate for the mean annual energy density in Table 6, where the underground safety distance applies^37^, i.e., we only consider the land use that cannot be used for other purposes. Nevertheless, this assumption neglects that stricter safety distances apply to pipelines in the vicinity of buildings^37^ and overlooks the pipeline’s right-of-way agreement distance, where certain activities are prohibited^38,39^.

**Table 4:** Mean and annual power and energy densities for combined-cycle natural gas power plants scatter-plotted in Fig. 2.

Mean specific power ($p_g$)		Annual energy density ($\varepsilon _g$)		
*mdn*	$avg \pm dev$	*mdn*	$avg \pm dev$	*n*
${2246.57}\,{\hbox {W/m}^2}$	2696.17 ± 1782.52 ${\hbox {W/m}^{2}}$	19.693 ${\hbox {TWh/km}^2}$	23.634 ± 15.625 ${\hbox {TWh/km}^2}$	26

**Table 5:** Percentage share of the land use intensity across several stages of the fuel cycle of natural gas based on a 500-site study in the Barnett Shale of Texas^13^.

Production sites	Gathering pipelines	Gathering sites	Processing sites	Transmission pipelines	Transmission sites	Power plants	Total
10.63%	80.12%	0.07%	0.38%	1.42%	0.02%	7.36%	100.00%

**Table 6:** Data needed to calculate the annual energy density including the land use of pipelines (final result at the bottom).

Total primary energy from natural gas (2020)^35^	38,611 TWh
Share of the total primary energy for electricity generation (estimation)	32.8 %
Total energy use for electric power generation (estimation)	12,666 TWh
Mean thermal efficiency of natural fleet (assumption)	50.0 %
Total electric power generation (2020)^35^	6333 TWh
Installed rated power (2020)^35^	1830 GW
Accumulated capacity factor (2020)	39.5 %
Mean pipeline diameter (assumption)$^{\text {a}}$	0.8 m
Lower pipeline safety distance (from other underground facilities)^37^	0.3m
Upper pipeline safety distance (from buildings)^37^	1.5 m
Right-of-way (ROW) pipeline agreement distance (certain activities prohibited)^38,39^	7.6 m
Lower / upper pipeline strip of land width ($\approx$ 2 $\times$ safety distance + pipeline diameter)	1.4 m/3.8 m
Total pipeline length worldwide (2016)$^{\text {b}}$	2,781,000 km
Lower / upper estimate of pipeline land use worldwide ($\approx$ strip width x pipeline length)	3893 ${\text{km}^2}$/10,568 ${\text{km}^2}$
Lower / upper estimate of pipeline land use for electricity generation (scaled by gas share)	1277 ${\text{km}^2}$/3466 ${\text{km}^2}$
Power plant land use for electricity generation (using the overall value in Table 4)	268 ${\text{km}^2}$
Fuel production land use (44% higher than that for the power plants, listed in Table 5)	386 ${\text{km}^2}$
Upper / lower estimate of natural gas land use for electricity generation (approximated)	1931 ${\text{km}^2}$/4120 ${\text{km}^2}$
Power plant mean annual energy density (excl. pipeline and production, retrieved from Table 4)	23.634 ${\text{TWh}/\text{km}^2}$
Lower estimate of mean annual energy density (approx. incl. pipelines and production pads)	1.537 ${{\text { TWh/km}}^2}$
Upper estimate of mean annual energy density (approx. incl. pipelines and production pads)	3.280 ${{\text { TWh/km}}^2}$

The assessment is based on 2020 thermal electricity generation from natural gas, where the length of natural gas pipelines is retrieved from available data^40^.

$^{\text {a}}$The pipeline diameter can range from 50.8 to 106.7 cm.

$^{\text {b}}$2,004,000 km in North America, 271,000 km in the Commonwealth of Independent States (CIS), 187,000 km in Europe, 168,000 km in Asia and Oceania, 69,000 km in Latin America, 41,000 km in the Middle East, and 41,000 km in Africa^40^.

### Nuclear power

**Figure 3:**
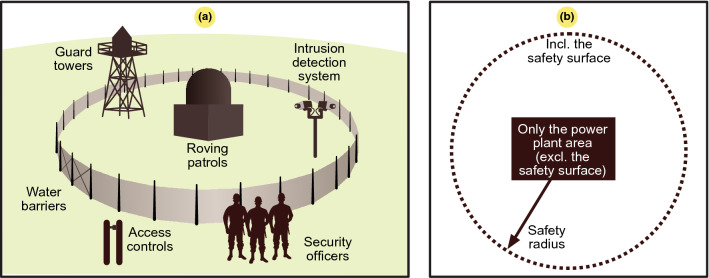
Security measures at a nuclear power plant to protect people and the environment in accordance with the United States Nuclear Regulatory Commission (USNRC). (**a**) Side view. (**b**) View from above indicating the safety radius including an exclusion area and a barrier space allocated in case of possible accidents.

The power and energy density of a nuclear power plant differ from those of a natural gas power plant, as there exist a unique additional land requirement, and the volume of nuclear fuel is significantly smaller. Indeed, as shown in Fig. 3a, a nuclear power plant requires a security zone that greatly contributes to increasing its space requirements. There occurs a protective barrier at the boundary where security personnel are located for control purposes. The estimated surface was obtained from Google Earth using the boundary defined based on the main safety radius, as this was clearly defined at the first barrier. A circular area is shown as an example of the safety surface of a nuclear power plant in Fig. 3b.

**Figure 4:**
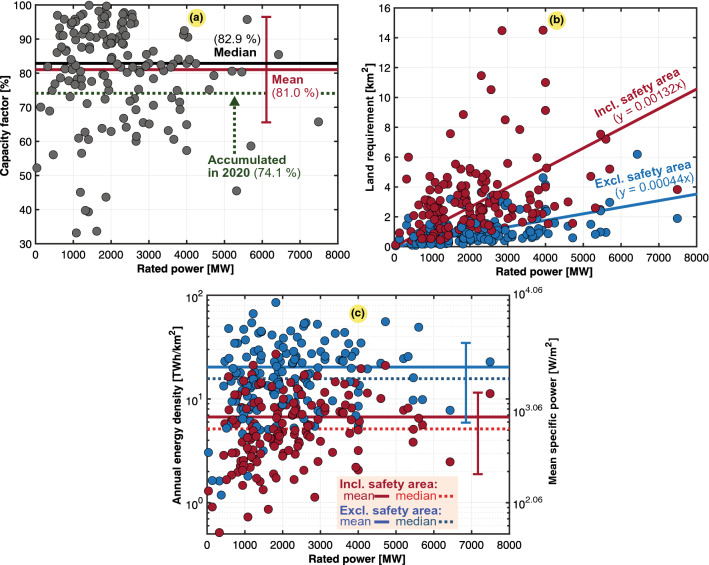
Scatter plots of the 159 nuclear power plants worldwide, 56 in Europe (1 in the Netherlands, 2 in Belgium, 19 in France, 6 in UK, 7 in Germany, 5 in Spain, 1 in Armenia, 2 in the Czech Republic, 2 in Finland, 1 in Hungary, 1 in Romania, 2 in Slovakia, 1 in Slovenia, 3 in Sweden, and 3 in Switzerland), 14 in the CIS (10 in Russia and 4 in Ukraine), 2 in the Middle East (1 in the United Arab Emirates and 1 in Iran), 1 in Africa (South Africa), 4 in Latin America (2 in Argentina, 1 in Brazil, and 1 in Mexico), 59 in North America (55 in the US and 4 in Canada), and 23 in Asia Pacific (4 in South Korea, 2 in Taiwan, 5 in Japan, 1 in the Philippines, 4 in China, 7 in India, and 2 in Pakistan). Typical, mean, median, and fitted values are also included. (**a**) Capacity factor ($C_g$). (**b**) Land use ($A_g$). (**c**) Annual energy density ($\varepsilon _g$) or mean specific power ($p_g$).

Scatter plots for the 159 nuclear power plants located in 32 countries considered in this study are shown in Fig. 4. To support the veracity of our findings, we performed an approximated capacity factor calculation of the worldwide nuclear power generation fleet in operation in 2020. Collectively, with a rated power of 393 GW, they generated 2553 TWh in total^41^. With the use of Eq. (1), we determined an accumulated capacity factor ($C_g$) of 74.1% for the whole fleet. Compared to the mean results of our population study, this value was of the same order of magnitude, with a deviation of only $+$6.9% ($C_g$ was 81%). Table 7 highlights that the energy density increases threefold when excluding the safety area. The mean annual energy density ($\varepsilon _g$) for the population was 6.703 ${{\text {TWh/km}}^2}$, including the safety surface, approximately twice the value of 3.333${{\text { TWh/km}}^2}$, as determined based on the land use per unit of electricity in a recent LCA study of power generation (within one standard deviation of our results). The LCA study was based on a different methodology with certain assumptions and data inputs to establish a generic case for a nuclear power plant^10,26^. The output highly depends on the assumptions made for associating the land use of waste storage or whether it is not stored above ground and can be neglected. Deep geological repository sites are expected as soon as 2023^10^. Regarding fuel extraction from 2016 to 2020, a significant portion was derived from underground mines (32%), while in situ leaching accounted for the majority (55%) of the total fuel extraction, whereas the most land-intensive open-pit mining occupied the minority (14%) of the total fuel extraction^10^.

**Table 7:** Mean and annual power and energy densities for nuclear power plants excluding and including the safety area (considering the data shown in Fig. 4).

	Mean specific power ($p_g$)		Annual energy density ($\varepsilon _g$)		
Boundary	*mdn* (${{\text {W/m}}^2}$)	$avg \pm dev$ (${{\text { W/m}}^2}$)	*mdn* (${{\text {TWh/km}}^2}$)	$avg \pm dev$ (${{\text {TWh/km}}^2}$)	*n*
Excl. safety area	1790.55	2313.94 ± 1640.67	15.696	20.284 ± 14.382	159
Incl. safety area	587.17	764.69 ± 549.69	5.147	6.703 ± 4.819	159

## Renewable resources

The majority of this paper is dedicated to renewable sources, which will be the focus of this section, divided into separate subsections.

### Geothermal power

**Figure 5:**
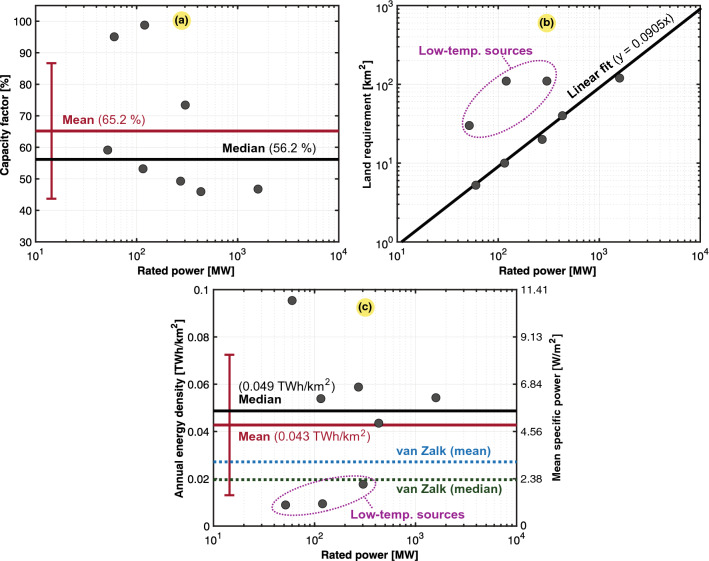
Case study of 8 geothermal power plants, 3 in Iceland and 5 in the United States. Typical, mean, median, and fitted values are also included. (**a**) Capacity factor ($C_g$). (**b**) Land use ($A_g$). (**c**) Annual energy density ($\varepsilon _g$) or mean specific power ($p_g$) with van Zalk and Behrens^3^ as a reference.

The results obtained for geothermal power generation were retrieved from a population study of 8 power plants located in Iceland and the US, which are two of the three most prominent countries (including Indonesia) in terms of geothermal energy produced. To determine the land required by geothermal power plants, we used the perimeter delimited by power plant wells, and we then estimated the surface area within this perimeter by using a satellite view in the Google Earth environment. The final results are shown in three scatter plots in Fig. 5. Based on our analysis, some power plants were close in terms of the calculated power and energy densities compared to those of van Zalk and Behrens^3^. However, our population included a larger portion of high-temperature generation facilities (temperature threshold $\ge$ 250 $^{\circ }C$) known to exhibit higher power densities, causing the mean power density to be approximately 58% higher than that of van Zalk and Behrens^3^.

### Hydropower

**Figure 6:**
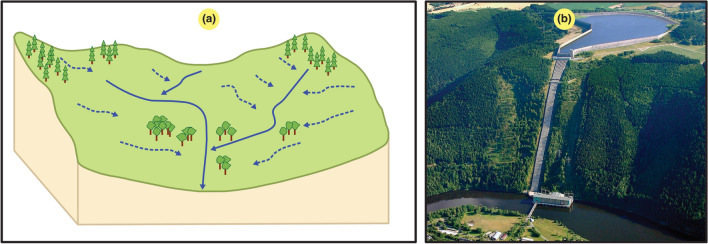
Illustration of the surface areas needed for hydroelectric power generation. (**a**) Catchment area (i.e., source area, $A_s$) used to collect the water. (**b**) Reservoir-allocated surface area ($A_g$) of the Hohenwarte II pumped-storage power plant (taken by Vattenfall on October 19, 2011, https://www.flickr.com/photos/vattenfall/6779452824, published under the courtesy of the CC BY-NC-ND 2.0 license, https://creativecommons.org/licenses/by-nc-nd/2.0/).

In this study, we particularly focused on large-to-small hydropower plants with a dam and a reservoir and not run-of-river hydropower plants. Land use definitions could vary between the catchment area (i.e., source area, $A_s$) and the reservoir surface area (i.e., generation area, $A_g$). Figure 6 shows the difference between these two definitions. In LCA, it is common to consider the inundated land area (ILA) of water surfaces while neglecting the construction of associated infrastructure^42^. However, the water body surface before dam construction is typically subtracted from the highest regulated water level, which yields a slightly lower ILA than the reservoir surface area considered in this paper.

In the literature, the mean power densities of hydropower plants could reach as low as $\le$ 1 ${{\text {W/m}}^2}$. The reason could be that the studied power plants exhibited a particularly low drop height, which could strongly influence the power and energy outputs. Another reason could be that the assumed area for the power density calculation was the catchment area. The ratio $A_s/A_g$ is a key metric to evaluate the difference between the source power and energy density ($p_s$ and $\varepsilon _s$) and the generation power and energy density ($p_g$ and $\varepsilon _g$), as formulated in Eq. (8) and originating from the concept of scaling.

8 $$\begin{aligned} p_g = p_{s} \frac{A_s}{A_g}, \quad \varepsilon _g = \varepsilon _{s} \frac{A_s}{A_g} \end{aligned}.$$

As shown in Fig. 6a, catchment areas ($A_s$) are locations in low-lying regions where water from higher areas accumulates into a single water body.

Reservoir-based hydropower facilities are built with their key conversion components underground, which hides them from the natural surface in their vicinity. This is the main argument when one considers the reservoir surface itself for power and energy density calculations. Contrary to Fig. 6b, there could occur hydropower plants with more than one reservoir occupying additional areas in which a very large amount of water could be stored. Therefore, we must carefully consider all reservoir surfaces to accurately determine $A_g$ in density calculations. For example, the hydropower plant in Saurdal, Norway, contains nine reservoirs to be considered in the calculation. It is also important to note that one reservoir could, in many instances, feed several hydropower plants, and therefore, they could share the assumed environmental footprint based on the solidarity principle, scaled by their respective energy output in energy density calculations.

**Figure 7:**
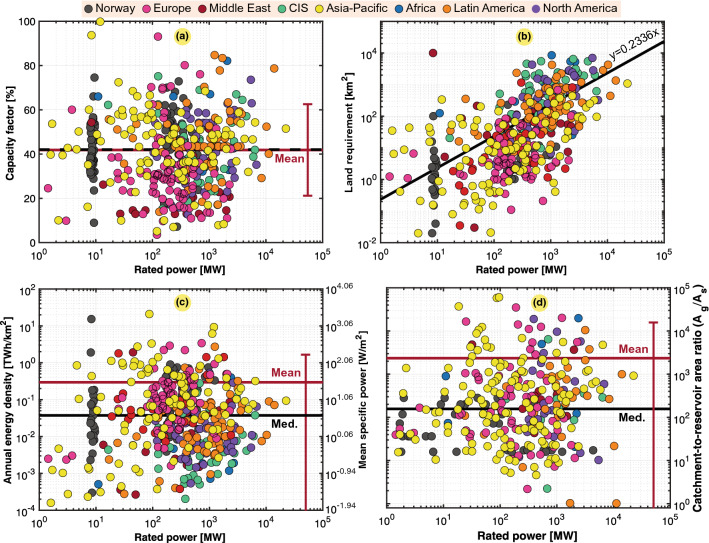
Scatter plots of 451 hydropower plants in the world, 71 in Norway, 68 in Europe, 21 in Middle East, 38 in the CIS, 98 in Asia Pacific, 12 in Africa, 34 in Latin America, and 26 in North America. Typical, mean, median, and fitted values are also included. (**a**) Capacity factor ($C_g$). (**b**) Land use ($A_g$). (**c**) Annual energy density ($\varepsilon _g$) or mean specific power ($p_g$). (**d**) Catchment-to-reservoir area ratio.

In our worldwide population study of hydropower generation, the catchment area ($A_s$) was obtained for 256 power plants, while 459 power plants were investigated based on the reservoir surface ($A_g$). Figure 7 shows scatter plots of the obtained and calculated data, where Fig. 7a–c show the capacity factor, land use of reservoirs ($A_g$) and power and energy density based on the considered land use, respectively. In many instances, hydropower dominates the power system, so in these cases, the load variation of the power system fed by hydropower more notably influences the capacity factor rather than representing a physical property of the power plant itself. Therefore, Fig. 7a shows that $C_g$ greatly varied. There was also a slight correlation between the land use and the rated capacity of hydropower plants, as shown in Fig. 7b. Wide variations in the power and energy density were observed, as shown in Fig. 7c, which are further quantified in Table 8 to reveal the differences between the various regions worldwide. The Asia Pacific region exhibited the highest density, at more than double that of Europe, which approached the overall density worldwide. Nevertheless, Europe exhibited outliers due to topography differences, and Norway was significantly denser than Europe as a whole.

As shown in Fig. 7d, we could observe why using the reservoir area ($A_g$) yielded a much higher power density than that based on the catchment area ($A_s$). Indeed, with our calculations, the ratio between the values based on these two surfaces reached 3040. Moreover, considering the median, we could obtain a factor of 208.

**Table 8:** Mean and annual power and energy densities for hydropower in the different regions.

	Mean specific power ($p_g$)		Annual energy density ($\varepsilon _g$)		
Region	*mdn* (${{\text {W/m}}^2}$)	$avg \pm dev$ (${{\text {W/m}}^2}$)	*mdn* (${{\text {TWh/km}}^2}$)	$avg \pm dev$ (${{\text {TWh/km}}^2}$)	*n*
Asia Pacific	9.03	75.11 ± 272.83	0.079	0.658 ± 2.39	98
Norway	7.52	45.71 ± 207.33	0.066	0.401 ± 1.817	71
Europe	8.94	33.73 ± 69.10	0.078	0.296 ± 0.606	92
Middle East	1.95	16.71 ± 48.00	0.017	0.147 ± 0.421	21
Latin America	1.95	10.98 ± 38.62	0.017	0.096 ± 0.339	72
North America	1.25	4.71 ± 10.27	0.011	0.041 ± 0.090	47
Africa	0.43	4.18 ± 13.02	0.007	0.037 ± 0.114	12
CIS	0.39	2.45 ± 6.05	0.003	0.021 ± 0.053	38
The World	4.26	33.73 ± 157.28	0.037	0.296 ± 2.379	451

It is unfortunate that hydropower, even though it often exhibits a very high power and energy density among renewables (depending on the region), is not as easy to scale up as other renewable sources. However, there remains a high potential for the retrofitting of existing dams, which is an emerging solution to introduce more hydropower in existing occupied reservoirs.

The above is a very important consideration, as it has been previously emphasized that dams exert a significant environmental impact. Indeed, an international survey of the 60,000 largest dams globally indicated that only one-third of all dams was used for hydropower production^43^. Even in Europe, Poland exhibited a significant unused hydroelectric potential^44^. Moreover, there are 90,000 dams in the US and only approximately 2500 of these dams generate power, i.e., $\sim$ 3%. While most of these nonpowered dams do not store enough water or are too remote to be suitable for hydropower development, thousands could be retrofitted to generate electricity, according to the US Department of Energy (DOE). In a 2016 report^45^, the agency found that retrofitting existing dams could add as much as 12,000 MW of generation capacity to the grid. Developing these dams could, therefore, yield a double effect, greatly reducing the environmental impact by utilizing existing constructions and increasing electricity production with hydraulic hydropower technology.

According to known hydropower data, the world’s production reached 4418 TWh in 2020^22^. Assuming that only one-third of the total dams is used, that we can use 100% of these dams to generate hydraulic energy and that they, on average, can be linearly scaled, we can obtain 13,254 TWh of electricity generation. This corresponds to approximately 50% of the global electricity generation in 2020. However, this result is utopian, as the International Energy Agency’s (IEA’s) prediction assumed only a 91.6% increase in hydropower toward 2050, which accounts for only 63.9% of our estimated potential. Nevertheless, hydropower generation could significantly contribute to the global energy mix by 2050.

### Solar power

Large solar power plants are either photovoltaic (PV) or concentrated solar power (CSP) plants, where the latter tends to exhibit a higher energy density.

**Figure 8:**
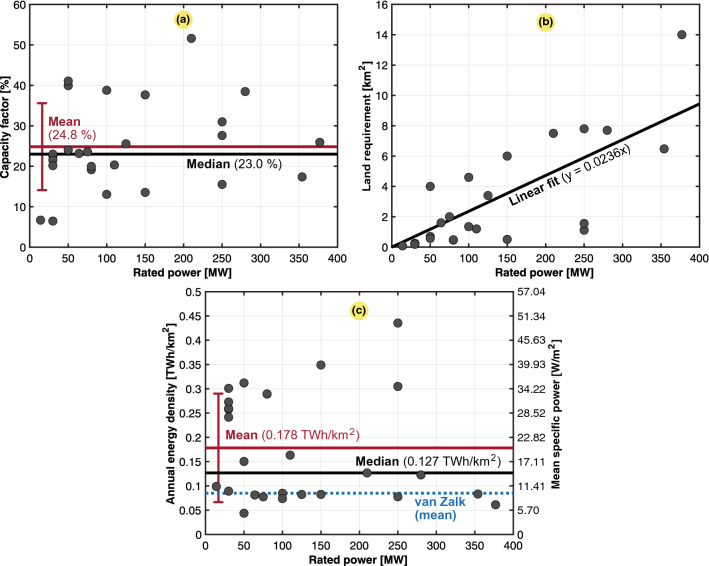
Case study of 27 concentrated solar power (CSP) plants, 17 in the United States, 7 in Spain, 1 in India, 1 in Chile, and 1 in South Africa. Typical, mean, median, and fitted values are also included. (**a**) Capacity factor ($C_g$). (**b**) Land use ($A_g$). (**c**) Annual energy density ($\varepsilon _g$) or mean specific power ($p_g$) with van Zalk and Behrens^3^ as a reference.

CSP plants were studied via a scatter plot of 27 large CSPs worldwide, as shown in Fig. 8. It should be noted that CSP farms usually attain a capacity factor between 15% and 30%^46^, even though expected industry values can reach as high as 50%. One standard deviation of the mean of the population depicted in Fig. 8a covers values ranging from 14.1% to 35.6%, which agrees with earlier reporting^46^. Even though the land use of CSP plants could be linearly fitted with respect to the rated capacity, a high deviation exists, as shown in Fig. 8b. This is also reflected in the power and energy density scatter plot of Fig. 8c, where our analysis yielded higher densities than those of van Zalk and Behrens^3^, which involved a more limited population study ($n = 2$).

Solar PV systems can be used in residential applications. Here, rooftop PV systems were selected as a reference for utility-scale onshore and offshore solar PV farms. Residential PV values were obtained from a recent study of up to 40 countries^5^. The rooftop PV data contained no installation specifications but rather provided numbers based on the total installed rated power in these countries (i.e., accumulated power and energy densities). Rooftop PV technology solves the challenge of land use by utilizing existing surfaces on buildings. However, this technology is limited in scalability, as building surfaces are limited. Table 9 provides the geographical differences in residential PV applications, which are assumed to exhibit a similar land use relative to that of utility-scale PV farms. It is clear that Africa exhibits the highest PV potential, even though Capelan studied only one country in this region^5^.

**Table 9:** Mean and annual power and energy densities of residential solar PV systems in the different regions based on the estimated cumulative data for each country^5^.

	Mean specific power ($p_g$)		Annual energy density ($\varepsilon _g$)		
Region	*mdn* (${{\text {W/m}}^2}$)	$avg \pm dev$ (${{\text {W/m}}^2}$)	*mdn* (${{\text {TWh/km}}^2}$)	$avg \pm dev$ (${{\text {TWh/km}}^2}$)	*n*
Africa (South Africa)	17.35	17.35 ± 0.00	0.152	0.152 ± 0.000	1
Latin America	9.00	9.00 ± 0.52	0.079	0.079 ± 0.005	2
Asia Pacific	7.83	7.05 ± 2.08	0.069	0.062 ± 0.018	7
Middle East	6.38	6.38 ± 1.43	0.056	0.056 ± 0.013	2
North America	4.78	5.58 ± 3.45	0.042	0.049 ± 0.030	3
Europe	3.70	3.83 ± 1.53	0.032	0.034 ± 0.013	29
CIS (Russia)	2.52	2.52 ± 6.05	0.022	0.022 ± 0.000	1
The World	4.17	4.58 ± 2.25	0.037	0.040 ± 0.020	40

**Figure 9:**
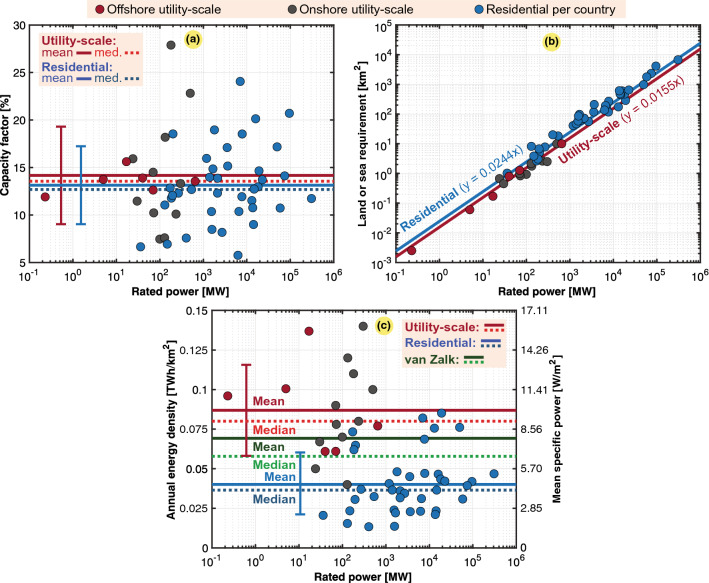
Scatter plots of onshore and offshore utility-scale PV farms and residential PV farms, namely, 11 onshore PV farms ($n= 11$), including 1 in Peru, 1 in South Korea, 1 in Japan, 1 in Taiwan, 1 in the Philippines, 1 in the Netherlands, 1 in Belgium, 1 in the UK, 1 in Germany, 1 in France, and 1 in Spain, and 6 offshore PV farms ($n= 6$), including 2 in France, 1 in China, 1 in India, 1 in Japan, and 1 in Singapore. Cumulative residential PV installations in 39 countries ($n= 39$), including 23 in Europe, 7 in Asia Pacific, 2 in the Middle East, 2 in Latin America, 2 in North America, and 1 in the CIS. Cumulative data retrieved from estimations^5^. Typical, mean, median, and fitted values are also included. (**a**) Capacity factor ($C_g$). (**b**) Land use ($A_g$). (**c**) Annual energy density ($\varepsilon _g$) or mean specific power ($p_g$) with van Zalk and Behrens^3^ as a reference.

Offshore solar PV farms provide several advantages over onshore installations. First, the water temperature approaches the equilibrium temperature of the solar panels. Second, large water bodies benefit from maximum sunlight and a clean environment. Finally, offshore PV technology allows the development of unexploited seas and can thus prevent possible space competition on land. However, this presupposes that offshore PV farms do not impact oceans as profoundly and negatively as do on-ground land installations. Nevertheless, offshore installations need more infrastructure to collect power and transfer it to land, which is associated with its own environmental footprint. Offshore PV farms remain less developed than onshore PV farms. However, an increasing number of floating solar plants are being built, but there is still a lack of data for these installations. This study was based on six offshore plants, while the onshore population involved eleven PV farms (Fig. 9).

In the scatter plots in Fig. 9, we distinguished the following three types of PV installations: utility-scale onshore and offshore installations and rooftop PV (residential) installations. Regarding utility-scale onshore and offshore PV farms, this study did not consider countries but actual large-scale solar farms. In general, larger PV farms are significantly more scalable in terms of their power and energy density, as highlighted in Fig. 9c. Even though onshore and offshore utility-scale PV farms are distinguished in the scatter plots, they are grouped in statistical analysis as they provide comparable energy densities.

### Wind power

Wind power exhibits, naturally, a relatively low capacity factor influencing its power and energy density. Wind turbines must also be distributed spatially to prevent negatively influencing each other’s performance, causing a lack of energy concentration. This section first evaluates the accumulated capacity factor of the global wind power fleet, as listed in Table 10. The global capacity factor in 2018 was 25.6%, while it reached 26.1% in 2019^24^, and 24.6% in 2020^24^. On average, the rated power of wind farms is available only one-fourth of the time, yielding the above accumulated energy.

**Table 10:** Wind power capacity factor for 2018 by country with the installed rated power retrieved from the Our World in Data database^47^.

	Total		Onshore (land-based)			Offshore (sea-based)		
Country	$P_g$ (MW)	$C_g$ (%)	$P_g$ (MW)	$E_g$ (GWh)	$C_g$ (%)	$P_g$ (MW)	$E_g$ (GWh)	$C_g$ (%)
China	184,665	22.6	180,077	357,340	22.6	4588	9112	22.7
USA	94,417	33.3	94,388	275,732	33.3	29	102	40.1
Germany	58,721	21.4	52,328	90,484	19.7	6393	19467	34.7
India	35,288	17.8	35,288	55,009	17.8	–	–	–
Spain	23,405	24.8	23,400	50,885	24.8	5	11	25.1
UK	21,768	29.8	13,551	30,217	25.4	8217	26687	37.1
France	14,900	21.9	14,900	28,599	21.9	–	–	–
Brazil	14,843	37.3	14,843	48,489	37.3	–	–	–
Canada	12,816	28.3	12,816	31,848	28.3	–	–	–
Italy	10,230	19.8	10,230	17,716	19.8	–	–	–
Sweden	7300	26.0	7097	15,953	25.6	203	670	37.7
Turkey	7005	32.5	7005	19,949	32.5	–	–	–
Denmark	6115	25.9	4414	9269	24.0	1701	4630	31.1
Poland	5766	25.3	5766	12,799	25.3	–	–	–
Australia	5679	30.5	5679	15,164	30.5	–	–	–
Portugal	5172	27.8	5172	12,617	27.8	–	–	-
Mexico	4875	30.1	4875	12,877	30.1	–	–	–
Netherlands	4393	27.4	3436	6934	23.0	957	3630	43.3
Ireland	3676	26.8	3651	8559	26.7	25	81	37.0
Japan	3667	23.3	3602	7376	23.4	65	105	18.4
Belgium	3268	26.1	2082	4154	22.8	1186	3311	31.8
Austria	3133	22.0	3133	6030	22.0	–	–	–
Greece	2877	25.0	2877	6300	25.0	–	–	–
South Africa	2094	35.2	2094	6467	35.2	–	–	–
Norway	1710	25.9	1708	3871	25.9	2	5	28.5
Chile	1524	26.9	1524	3588	26.9	–	–	–
Morocco	1225	35.9	1225	3856	35.9	–	–	–
Egypt	1125	23.7	1125	2334	23.7	–	–	–
New Zealand	784	30.1	784	2068	30.1	–	–	–
Argentina	750	21.5	750	1413	21.5	–	–	–
Ukraine	621	25.7	621	1399	25.7	–	–	–
Iran	282	23.7	282	585	23.7	–	–	–
Kazakhstan	122	43.1	122	461	43.1	–	–	–
Russia	52	16.9	52	77	16.9	–	–	–
Saudi Arabia	3	19.0	3	5	19.0	–	–	–
The World	563,830	25.6	540,204	1,194,718	25.2	23,626	68,196	32.9

**Figure 10:**
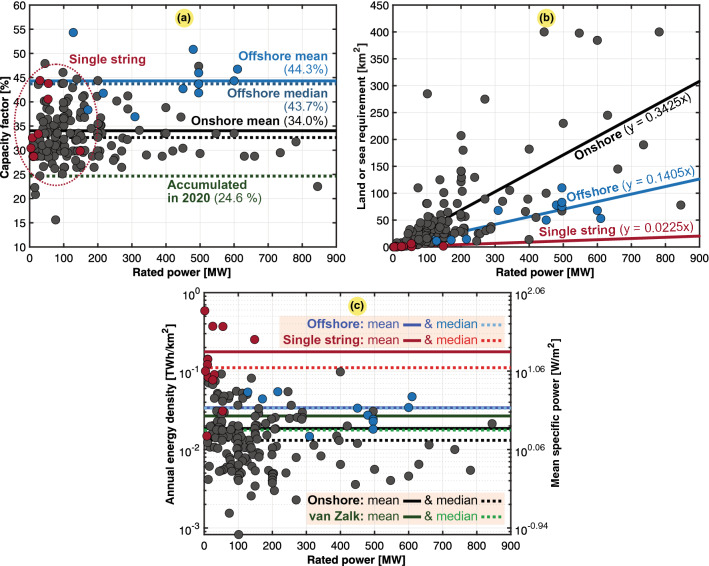
Worldwide study of 173 wind farms ($n = 173$), where 148 are onshore regular farms (4 in Africa, 3 in Latin America, 77 in North America, 53 in Europe, and 11 in Asia Pacific), 14 are single-string farms, and 11 are offshore farms. Typical, mean, median, and fitted values are also included. (**a**) Capacity factor ($C_g$). (**b**) Land use ($A_g$). (**c**) Annual energy density ($\varepsilon _g$) or mean specific power ($p_g$) with van Zalk and Behrens^3^ as a reference.

Regarding the power and energy densities, it is slightly more complicated to gather precise data. The total land use of a wind power plant comprises the area within the perimeter surrounding all turbines. A detailed investigation was conducted of each wind farm to identify the accurate amount of regulated land required for wind turbines ($A_g$). We were limited to Google Earth-obtained measurements. Our overall population study was based on 173 wind farms located in 19 countries, including 162 onshore wind farms, 14 single-string wind farms, and 11 offshore wind farms. The key data obtained for the different continents are listed in Table 11, which summarizes the onshore wind farm findings. The scatter plots in Fig. 10 also include offshore wind farms for comparison.

**Table 11:** Mean and annual power and energy densities for regular onshore wind power on the different continents, single-string onshore wind power, and offshore wind power.

Category	Mean specific power ($p_g$)		Annual energy density ($\varepsilon _g$)		*n*
	*mdn* (${{\text {W/m}}^2}$)	$avg \pm dev$ (${{\text {W/m}}^2}$)	*mdn* (${{\text {TWh/km}}^2}$)	$avg \pm dev$ (${{\text {TWh/km}}^2}$)	
Onshore					
Asia Pacific	0.90	1.19 ± 0.66	0.008	0.010 ± 0.006	11
North America	0.96	1.07 ± 0.61	0.008	0.009 ± 0.005	77
Europe	3.17	3.90 ± 2.48	0.028	0.034 ± 0.022	53
Latin America	2.39	2.51 ± 0.29	0.021	0.022 ± 0.003	3
Africa	0.83	1.03 ± 0.94	0.007	0.009 ± 0.008	4
The World	1.49	2.12 ± 2.06	0.013	0.019 ± 0.018	148
Single string	12.61	20.11 ± 18.56	0.110	0.176 ± 0.163	14
Offshore	3.84	3.89 ± 1.61	0.034	0.034 ± 0.014	11

Onshore wind installations were divided into the regular type and single-string topology, where the latter is a unique onshore case. In the string-based farm topology, no other wind turbines can disturb the operation of the installed wind turbines since they are arranged in a single row and not in columns, which leads to an improved performance. It is also ideal when geographical areas do not allow widely distributed wind farms, as they occupy only long, narrow areas (e.g., atop a mountain, along an ocean, etc.). Nevertheless, the main bottleneck for string-configured wind farms is that they are spatially not scalable because they do not allow turbine distribution along both directions horizontally, i.e., also along the wind direction.

We found that string-type wind farms attained a higher spatial energy density due to their inherently small spatial area regulated for installation. The estimated area is equivalent to a thin and straight corridor with the same width as the rotor diameter and projected along the entire string of turbines. Even though thin corridors led to a nearly tenfold reduction in land requirements, it remained an order of magnitude larger than the direct land use associated with the wind turbine towers and their corresponding infrastructure. According to the United Nations Economic Commission for Europe (UNECE)^10,26^, based on the direct land impact, a local annual energy density of approximately 2.50 ${{\text {TWh/km}}^2}$ was estimated. However, including indirect impacts and the total area needed, 0.01 ${{\text {TWh/km}}^2}$ was found for the regular wind farm topology^26^. The National Renewable Energy Laboratory (NREL) distinguishes between the directly impacted area and the total regulated area in their classification^31^. From the perspective of the total area, the straight corridor we assumed in this paper could be justified. Inevitably, a permanent service road and a temporary road occur on each side of the string of turbines^31^. We presumed that undisturbed land did not occur within this corridor. Nevertheless, the estimated width of the string corridor is a sensitive assumption even though it is found to scale well with the rotor diameter. The exact scaling could vary among different string-based wind farms, but we found a proportionality to the diameter that could provide a fairly satisfactory approximation. Via dimensional analysis, it is beneficial to consider a large rotor diameter to enhance the energy density. Size effects could reduce the environmental impact per TWh of production. It has been found this can be reduced by 14% for every doubling in the rated power (i.e., a larger rotor diameter increases power more than the land area needed), making the case for wind farms comprising larger turbines^48^.

According to the NREL^31^, the average spatial power density of regular land-based wind farms in the US is approximately 3 ${{\text {W/m}}^2}$, which occurs within one standard deviation of our data. It is also acknowledged that at high scales of deployment, the power density is reduced. In our data, a higher mean power density of offshore wind turbines (3.89 ${{\text {W/m}}^2}$) was determined than that of onshore facilities (2.12 ${{\text {W/m}}^2}$), as shown in Fig. 10c. This occurs because the areas are windier, and therefore, the capacity factor for offshore wind turbines is slightly higher (also found for the accumulated $C_g$ values in Table 10). However, offshore wind farms need more grid infrastructure to collect and transport energy from sea to land, where long-distance direct current grids are more relevant. Additionally, higher maintenance needs and a lower mobility at sea suggests higher operational costs. For these reasons, offshore wind is still not very widely developed today, but it remains very promising for the future, and the price has already significantly decreased in recent years.

**Figure 11:**
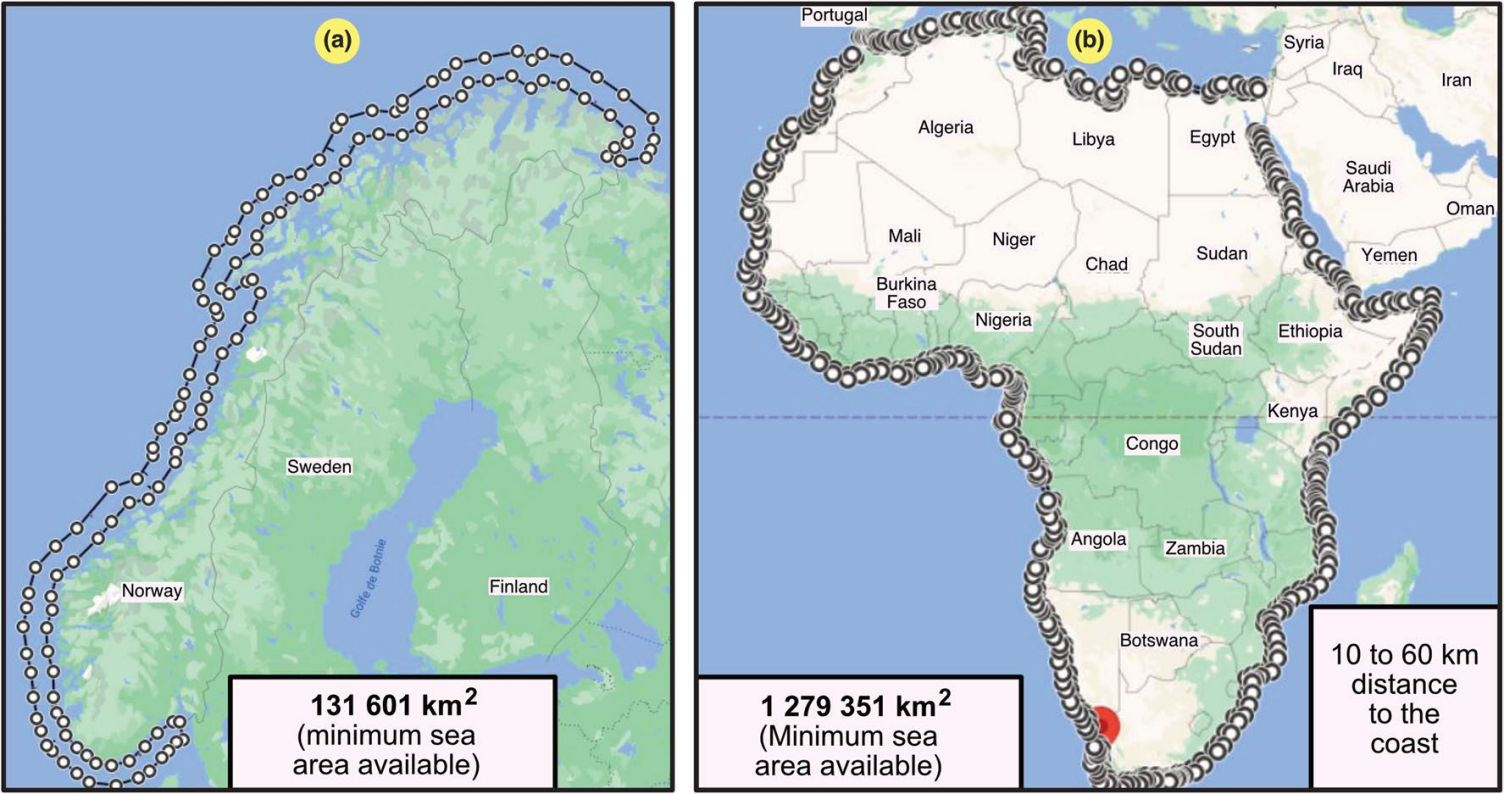
Illustrative examples to estimate the minimum available sea area (conservative case) for offshore wind turbines considering a corridor of just 50 km (more cases are provided in Table 13). They were created with the use of the Google Earth Engine^25^, version 7.3.2.5776 (https://earth.google.com/web/). (**a**) Norway. (**b**) Africa.

Offshore wind technology occupies space, but it needs sea area, not land area. When studying the occupied space of conventional offshore turbines, it is important to emphasize that they cannot be positioned just anywhere in the ocean. A useful area must be clearly defined. To not excessively occupy territorial waters, the shortest distance from the coastline for larger offshore windfarm projects is often 10 km^49^, where winds are stronger and more consistent. People living on the coast tend to not want to see large wind turbines, which is referred to as the not in my backyard (NIMBY) phenomenon. Table 12 provides positioning data available for 112 operative and 53 planned offshore windfarms. The mean distance from the shore in planned projects is approximately 30 km with a standard deviation of approximately 29 km. Based on these data, considering the abovementioned limitations near the coast, one could assume a majority of offshore windfarms to be placed within a corridor between 10 km 60 km from the shore. This is also the stricter limitation for conventional turbines sensitive to the sea depth. In the conservative estimate in this paper, offshore turbines were located within a coastal corridor approximately 50 km wide, between 10 km and 60 km from the coast. In addition to this conservative constraint, all the unavailable areas restricting this generic corridor are usually not easy to identify when focusing on a worldwide assessment not limited to certain regions. To illustrate the implications of the conservative estimates of useful areas, Fig. 11 shows two examples: Norway and Africa. According to Fig. 11a, Norway exhibited a total useful area of ${131{,}601}\,\,{\text{km}^2}$, which could be validated considering that Norway has a perimeter of 2632 km where the 50-km corridor applies. Similarly, Africa exhibited a total useful area of ${1{,}279{,}351}\,\,{\text{km}^2}$, corresponding to a perimeter of 25,587 km.

**Table 12:** Distance from the coastline among 112 operative and 53 planned offshore windfarms investigated in a 2020 offshore wind technology review^49^.

Category	*mdn* (km)	$avg \pm dev$ (km)	*max* (km)	*n*
Operative	10	18.85 ± 22.89	115	112
Planned	22	29.69 ± 28.69	120	53

To reveal the full potential of the spatial installation of offshore wind turbines, including new technologies such as floating wind farms, another more optimistic corridor limited by the exclusive economic zone (EEZ) should instead be considered^50^, which is a maritime area in which a coastal state has sovereign and economic rights to explore and use natural resources. Indeed, the EEZ area is a close estimate of the maximum useful area for offshore wind technology. In some countries, this area extends to a maximum of 200 nautical miles (NM) (370.42 km) from the coastline, before entering into international territory. A first-order approximation of the 100% energy from offshore wind scenario was made based on the described lower and upper estimates of the available sea area for the different countries and regions, as summarized in Table 13. Belgium exhibited a required area that was more than six times larger than the available area based on the conservative estimate. This occurred because Belgium has a short coastline directly impacting the coastline of the UK. France also faces challenges in meeting all its energy needs from offshore wind, but if approximately two-thirds of the 50-km corridor could be utilized, these needs could be met. However, this could challenge use of the coastline for other purposes, e.g., the maritime traffic could be disturbed.

**Table 13:** Defined 100% offshore wind scenario according to the 2020 energy use for the different handpicked countries or regions based on an offshore wind annual energy density ($\varepsilon _g$) of 0.034 ${{\text {TWh/km}}^2}$ (mean value of the offshore wind population in Table 11). The required sea area is calculated with Eq. (6) and compared relatively to the upper and lower estimates of the available sea area.

Region	Energy demand ($E_g \approx E_c$) (TWh)	Maximum sea area available$^{\text {a}}$ ($A_{max}$) (${\text{km}^2}$)	Minimum sea area available$^{\text {b}}$ ($A_{min}$) (${\text{km}^2}$)	Sea area required$^{\text {c}}$ ($A_g = E_g/\varepsilon _g$) (${\text{km}^2}$)	Optimistic area ratio ($A_{g}/A_{max}$) (%)	Conservative area ratio ($A_g/A_{min}$) (%)
Belgium	608	3447	2938	17,882	518.77	608.65
France	2418	371,096	104,797	71,118	19.16	67.86
Lebanon	107	19,516	9676	3147	16.13	32.52
Vietnam	1136	417,663	126,485	33,412	8.00	26.42
Georgia	74	21,946	12561	2176	9.92	17.32
Portugal	259	327,667	46,140	7618	2.32	16.51
Ecuador	179	450,000	39,345	5265	1.17	13.38
Latin America	7275	19,221,771	1,796,000	213,971	1.11	11.91
Africa	5167	12,774,090	1,279,351	151,971	1.19	11.88
Chile	444	2,017,717	241,167	13,059	0.65	5.41
Norway	211	1,273,482	131,601	6206	0.49	4.72
Yemen	47	552,669	73,112	1382	0.25	1.89
Iceland	57	751,345	91,088	1676	0.22	1.84
Liberia	5	249,734	34,278	147	0.06	0.43

$^{\text {a}}$Optimistic estimate of the available sea area ($A_{max}$), in the exclusive economic zone (EEZ), up to 370 km off the coastline.

$^{\text {b}}$Conservative estimate of the available sea area ($A_{min}$), defined as a 50-km ocean corridor from 10 to 60 km off the coastline.

$^{\text {c}}$Sea area that must be regulated for offshore wind technology to meet 100% of the energy demand.

### Tidal power

**Figure 12:**
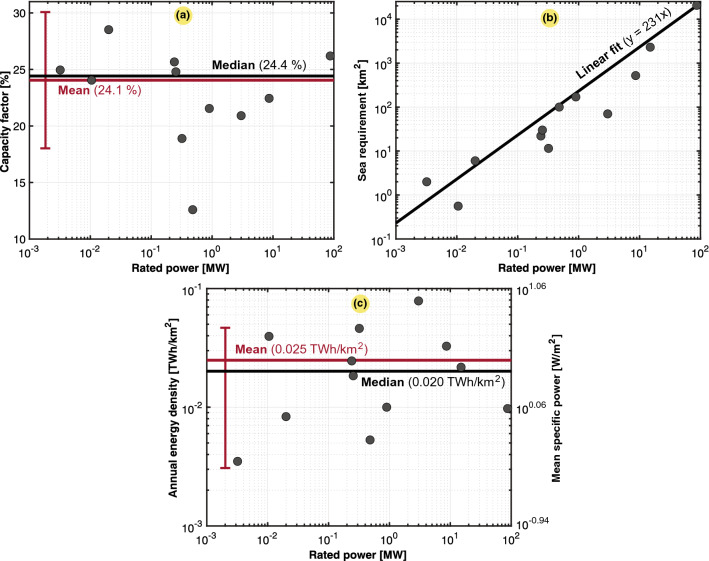
Case study of 12 tidal power plants worldwide ($n = 12$), 2 in the CIS (Russia), 1 in North America (Canada), 5 in Europa (1 in France and 4 in the UK), and 4 in Asia Pacific (1 in China, 1 in India, and 2 in South Korea). Typical, mean, median, and fitted values are also included. (**a**) Capacity factor ($C_g$). (**b**) Land use ($A_g$). (**c**) Annual energy density ($\varepsilon _g$) or mean specific power ($p_g$).

Tidal energy is not yet widely used, so there are few farms to collect data on. The results obtained for the tidal energy density are retrieved from a case study of 12 farms located in seven countries. The details are described in the caption of Fig. 12. The scatter plots in Fig. 12 include the capacity factor, pool land use, and power and energy density. Concerning the energy density, we found the same problem as that for hydropower, where two different surfaces could be considered. Either the area of the entire infrastructure (i.e., river, dam, and pool) or that of the pool only. In all calculations, the lower area estimate of the pool was considered. We found a mean power density of 2.84 ${{\text {W/m}}^2}$, or an annual energy density of 0.025 ${{\text {TWh/km}}^2}$. In fact, few power density studies of tidal energy have been conducted thus far. For comparison, another study found an average power density of 3 ${{\text {W/m}}^2}$ via calculation^33^, equivalent to an annual energy density of 0.026 ${{\text {TWh/km}}^2}$, which verifies the accuracy of our findings. Other studies only consider the swept area of the tidal turbine^51–53^, where estimates of the tidal stream power density become incomparable to this work.

Several sources estimated that the technically exploitable tidal power worldwide, in near-shore areas, is approximately 1 TW^54^, which corresponds to the production of roughly 8760 TWh per year. The world’s electricity consumption in 2020 was 22,492 TWh. Tidal energy could, therefore, only satisfy approximately 37% of the global electricity demand. As the world’s primary energy needs in 2020 reached 160,318 TWh, the exploitable tidal potential could only meet 5% of the demand. Even under the best-case scenario, if all the tidal energy were exploited, this would not meet the total consumption. This is a limitation in tidal power scaling, which should be regarded as a supplemental energy source.

### Wave power

**Figure 13:**
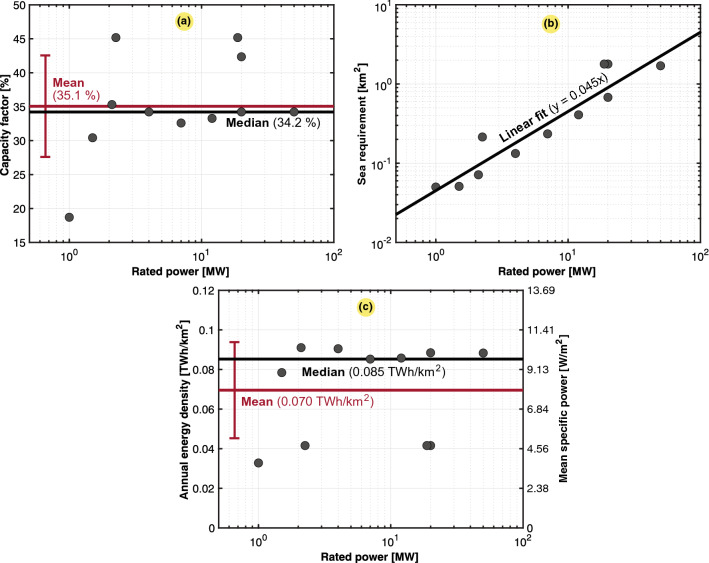
Case study of 11 prototype or designed wave power plants worldwide ($n = 11$), 1 in Scotland, 2 in Portugal, 2 involving Wave hub, 4 involving Wave dragon, 1 involving AWS, and 1 involving the Wave dragon wave generator. Typical, mean, median, and fitted values are also included. (**a**) Capacity factor ($C_g$). (**b**) Land use ($A_g$). (**c**) Annual energy density ($\varepsilon _g$) or mean specific power ($p_g$).

Wave energy provides a very high untapped potential, as approximetaly 10% of the world’s electricity consumption could be covered by wave generation, corresponding to a technically exploitable potential of 1400 TWh per year. However, several constraints slow the development of wave energy. First, the difficult environment (e.g., storms) could quickly damage power conversion equipment. Moreover, this technology exhibits a relatively high levelized cost of energy (LCOE). In addition, acceptability and social perception could pose problems (e.g., disturbance of fauna, marine traffic, and fishing). These constraints are the reasons why wave energy is not yet very widely developed. The population study in this paper was based on prototypes of different technologies already installed. We also considered planned future wave power projects to be developed in the coming years (e.g., the Pelamis converter, CETO converter, Waveswing system, Wave dragon, etc.). Our population study consequently considered the available data obtained from three prototype designs and eight future wave power projects. Figure 13 shows three scatter plots of the available data, including the expected capacity factor, allocated land use, and estimated annual energy density or mean specific power.

### Hybrid renewable power

Even though currently underdeveloped, hybrid renewable solutions combining solar PV and wind installations are promising for the future. They have been extensively studied, but just a few have already been installed^4,55^. Three hybrid parks were studied herein: Haringvliet Zuid (Netherlands), Parc Cynog (UK), and Port Augusta (Australia). These farms contain two parts: one part containing the hybrid solution, where PV panels are installed at the foot of wind turbines, and one part containing only wind turbines. We were mainly interested in the hybrid part of these projects. The area of the hybrid part was estimated using the Google Earth environment, and the final results are listed in Table 14. Notably, the hybrid solution revealed a significant increase in the hybrid annual energy densities. Wind power exhibits a structurally lower power density than that of solar PV technology. For this reason, the mean hybrid energy density of the three farms was approximately 53% higher than that of solar PV technology alone. Furthermore, the cost of these installations was reduced as the same grid connection could be used for both sources. Hybrid renewable parks are relevant in regions where land or sea areas with natural resources are scarce.

**Table 14:** Occupied areas and mean power densities of three hybrid renewable farms ($n = 3$) combining solar PV and wind technologies, including 1 in the Netherlands (Haringvliet Zuid), 1 in the UK (Parc Cynog), and 1 in Australia (Port Augusta).

	Hybrid gen. ($E_g$) (GWh)	Hybrid area ($A_g$) (${\text{km}^2}$)	Solar PV ($\varepsilon _{g,1}$) (${{\text {TWh/km}}^2}$)	Wind ($\varepsilon _{g,2}$) (${{\text {TWh/km}}^2}$)	Hybrid total ($\varepsilon _g$) (${{\text {TWh/km}}^2}$)
Hybrid farm 1	8.73	0.09	0.075	0.022	0.097
Hybrid farm 2	93.72	0.44	0.128	0.085	0.213
Hybrid farm 3	258.40	6.80	0.024	0.014	0.038
Mean value	120.28	2.44	0.076	0.040	0.116

## Analyzing the obtained energy densities for land and sea use

This section finalizes the analysis of the nonrenewable and renewable resources investigated in the two previous sections. Here, we considered the energy consumption today and the predicted electricity needs in the future to obtain estimates of the needed surface area for the global generation mix.

In grouping the worldwide energy consumption into specific regions, we could establish the local annual energy density of the energy consumption, as listed in Table 15. These values could then be used to estimate the land and sea requirements of the different energy sources in the region to match the specific consumption pattern. Since the annual energy density of consumption is based on the primary energy use, this value could be adopted as the upper estimate of the potential electric energy use in the different regions. This is based on the assumption that deep decarbonization causes an increase in the electrification level of the energy supply. However, this transition also significantly increases the efficiency of energy use^30^. Primary energy is, therefore, used as an upper estimate of the future all-purpose electric energy demand.

**Table 15:** Mean specific power and annual energy density of consumption in the different continents and regions in the world, based on a 2020 scenario^22,56^, sorted by the consumption density.

Continents	Specific power ($p_c$) (${{\text {W/m}}^2}$)	Energy density ($\varepsilon _c$) (${{\text { GWh/km}}^2}$)	Consumption ($E_{c}$) (TWh)	Total area ($A_{tot}$) (${\text{km}^2}$)
Asia Pacific	0.287	2.512	70,347	28,000,000
Europe	0.240	2.105	21,430	10,180,000
Middle East	0.160	1.404	10,123	7207,575
North America	0.138	1.213	29,972	24,710,000
CIS	0.058	0.507	10,320	20,368,759
Latin America	0.043	0.379	7275	19,200,000
Africa	0.019	0.170	5162	30,370,000
The World (land area only)	0.125	1.095	163,056	148,939,063
The World (w/oceans)	0.036	0.320	163,056	510,100,000

The calculations are based on Eqs. (2) and (4), respectively.

**Figure 14:**
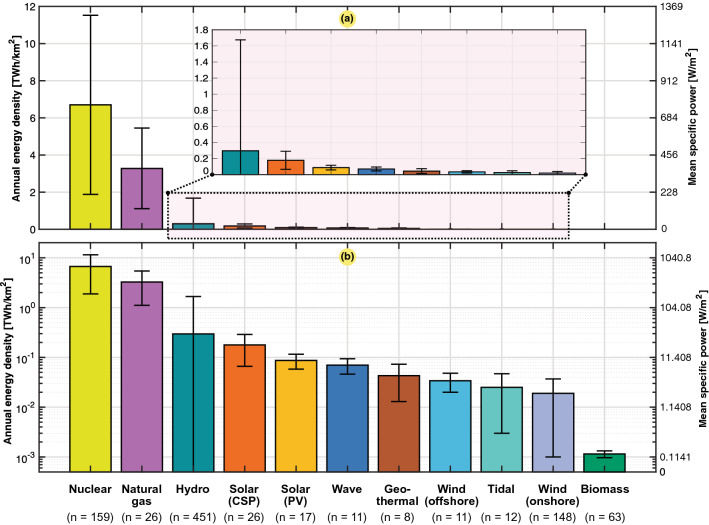
Overall result of the average values and standard deviations of the annual energy density ($e_g$) or mean specific power ($p_g$) for the different energy sources considered based on the output data provided in Table 16 (rooftop solar PV technology is excluded). (**a**) Linear scale. (**b**) Logarithmic scale. The biomass numbers are based on the meta-analysis of van Zalk and Behrens^3^.

Similar to the power consumption, the annual energy densities of the power sources are summarized in Table 16 as aggregated values, including respective standard deviations. The same values are plotted on linear and logarithmic scales in Fig. 14. The obtained aggregated data establish metrics of each source used in land use estimation hereafter.

The mean values in Table 17 of the annual energy density in this work were compared to other results reported in the literature. Even though there were significant deviations from our population studies, our mean results remained on the same order as that of the findings in other studies. One outlier was that the mean value of natural gas obtained from van Zalk and Behrens^1^ was overestimated, occurring outside one standard deviation in our work. Moreover, the solar CSP and PV values obtained from the UNECE^10^ and OWID^26^ were slightly below one standard deviation of our mean values.

**Table 16:** Aggregated result of the mean and annual power and energy densities of the 10 different energy resources examined in this paper (cumulative rooftop solar PV technology per country is separated in this table), referring to the data in Figs. 2, 4, 5, 7, 8, 9, 10, 12, and 13.

	Mean specific power ($p_g$)		Annual energy density ($\varepsilon _g$)		$C_g$	
Source	*mdn* (${{\text {W/m}}^2}$)	$avg \pm dev$ (${{\text {W/m}}^2}$)	*mdn* (${{\text {TWh/km}}^2}$)	$avg \pm dev$ (${{\text {TWh/km}}^2}$)	*avg* (%)	*n*
Nuclear	587.17	764.69 ± 549.69	5.147	6.703 ± 4.819	81.0	159
Natural gas	350.37	374.14 ± 247.38	3.071	3.280 ± 2.168	52.7	26
Hydro	4.26	33.73 ± 157.28	0.037	0.296 ± 1.379	41.9	451
Solar (CSP)	14.45	20.33 ± 12.74	0.127	0.178 ± 0.112	24.8	27
Solar (PV)	9.13	9.91 ± 3.28	0.080	0.087 ± 0.029	14.2	17
Wave	9.73	7.94 ± 2.77	0.085	0.070 ± 0.024	35.1	11
Geothermal	5.56	4.88 ± 3.39	0.049	0.043 ± 0.030	65.2	8
Solar (rooftop)	4.17	4.58 ± 2.25	0.037	0.040 ± 0.020	13.1	39
Wind (offshore)	3.84	3.89 ± 1.61	0.034	0.034 ± 0.014	44.3	11
Tidal	2.29	2.84 ± 2.49	0.020	0.025 ± 0.022	24.1	12
Wind (onshore)	1.49	2.12 ± 2.06	0.013	0.019 ± 0.018	34.0	148
Biomass^3^	0.08	0.13 ± 0.02	0.001	0.001 ± 0.000	n.a.	63

The mean, median and standard deviation are given, as well as the average capacity factor and the number of samples (*n*) per population study. The biomass numbers are based on the meta-analysis of van Zalk and Behrens^3^.

**Table 17:** Comparison of the annual energy densities of the different power sources found in the literature and comparison to the mean values found in the population studies (please refer to Table 16) of this work.

Source	Smil^1^ (${{\text {TWh/km}}^2}$)	van Zalk and Behrens^3^ (${{\text {TWh/km}}^2}$)	UNECE^10^ and OWID^26^ (${{\text {TWh/km}}^2}$)	This work (${{\text {TWh/km}}^2}$)
Nuclear	7.012–21.038	2.532	3.333	6.703
Natural gas	1.753–17.532	11.254	1.000	3.280
Hydro	0.001–4.383	0.003	0.071	0.296
Solar (CSP)	0.035–0.088	0.085	0.045	0.178
Solar (PV)	0.035–0.079	0.051	0.063	0.087
Geothermal	$\le$ 0.280	0.027	n.a.	0.043
Wind (offshore)	n.a.	0.037	n.a.	0.034
Wind (onshore)	0.004–0.013	0.027	0.010	0.019

The other studies also represent the generation facility’s spatial extent via the total area requirement. Smil provides lower and upper limits, while van Zalk and Behrens, United Nations Economic Commission for Europe (UNECE) and Our World in Data (OWID) specify mean values.

Table 18 provides the spatial requirements to achieve 100% primary energy in the different regions based on the various power sources examined in this paper. In general, it could be observed that nuclear power needed the smallest amount of space in each region, while biomass needed the largest amount of land. In the world as a whole (land areas only), the land use ranged from 96.154% land to as low as 0.016%. All the investigated sources were distributed within this window. Please note that regarding marine energy, such as offshore wind, tidal, and wave energy, the needed area was calculated in terms of land areas for the sake of comparability to that of the other sources (i.e., their minimum or maximum available areas were not considered). It is also important to note that the estimations were based on only the annual energy densities of the sources. The estimates did not consider that some sources faced limitations regarding the available areas that are strictly region-specific (e.g., hydropower).

**Table 18:** Spatial requirements for the different power sources to meet 100% of the primary energy use, normalized by the world’s land area, based on Eq. (5), where $A_g/A_{tot} \approx p_c/p_g = \varepsilon _c/\varepsilon _g$. The 2020 energy use (provided in Table 15) and the mean power density of the energy sources given by region or globally are listed in Tables 8, 11, and 16. It is assumed that all areas are amenable to each source and that they face no limitations in terms of scalability other than their mean annual energy densities.

Region	Asia-Pacific (%)	Europe (%)	Middle East (%)	North America (%)	CIS (%)	Latin America (%)	Africa (%)	The World (%)
Nuclear	0.038	0.031	0.021	0.018	0.008	0.006	0.002	0.016
Natural gas	0.077	0.064	0.043	0.037	0.016	0.011	0.005	0.033
Hydro$^{\text {a}}$	0.382	0.712	0.958	2.930	2.367	0.392	0.455	0.371
Solar (CSP)	1.412	1.181	0.787	0.679	0.285	0.212	0.093	0.615
Solar (PV)	2.896	2.422	1.615	1.393	0.585	0.434	0.192	1.261
Wave$^{\text {b}}$	3.615	3.023	2.015	1.738	0.730	0.542	0.239	1.574
Geothermal	5.881	4.918	3.279	2.828	1.189	0.881	0.389	2.561
Solar (rooftop)	3.665	6.486	2.508	2.887	2.302	0.478	0.110	2.998
Wind (offshore)$^{\text {b}}$	7.378	6.170	4.113	3.548	1.491	1.105	0.488	3.213
Tidal$^{\text {b}}$	10.106	8.451	5.634	4.859	2.042	1.514	0.669	4.401
Wind (onshore)	24.118	6.154	7.547	12.897	2.736	1.713	1.845	5.896
Biomass$^{\text {c}}$	220.769	184.615	123.077	106.154	44.615	33.077	14.615	96.154

$^{\text {a}}$The energy density of hydro power depends on the region and is constrained by natural resources, limiting the scalability.

$^{\text {b}}$The required sea area for wave, tidal and offshore wind farms is normalized by the total land area for comparability to other sources. Offshore wind considers a separate 100% scenario based on the available sea area in Table 13.

$^{\text {c}}$The biomass scenario uses the meta-analysis of van Zalk and Behrens^3^.

To facilitate an even more detailed-level assessment of the land and sea requirements for electric power generation, Table 19 lists the needed electricity mix toward 2050 to reach the NZE target based on the predicted growth in energy use^22^. This mix is based on the NZE normative scenario of the IEA, which provides a narrow but assumed achievable pathway to net zero emissions by 2050. The NZE roadmap assumes slightly below a threefold increase in worldwide electric energy use by 2050. Examples of electrification predictions indicate that 60% of all car sales will comprise electric vehicles (EVs) by 2030 and that 50% of sold heavy trucks will comprise electric vehicles by 2035. Moreover, electricity is expected to achieve net zero emissions in advanced economies by 2035 and globally by 2040.

**Figure 15:**
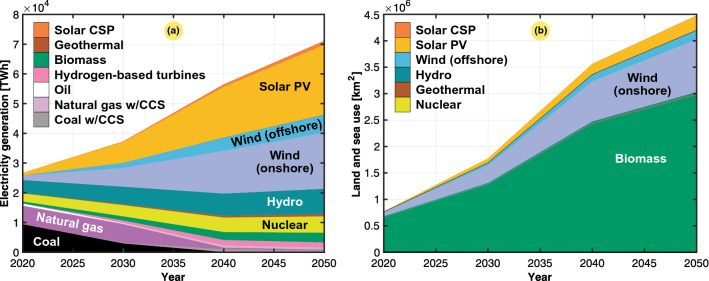
Filled area plots of the changes in the worldwide energy mix composition according to the IEA NZE scenario^22^. (**a**) Electricity generation mix (adopted from Table 19). (**b**) Predicted land and sea use requirements (adopted from Table 20).

The resulting land and sea use needed to achieve the IEA NZE predictions listed in Table 19 are given in Table 20. With the use of the mean energy density values in Table 16, the needed land and sea uses were calculated for the different sources ($A_g$) via Eq. (6). Each spatial component was summed to determine the total area of land needed, including and excluding sea use. It was demonstrated that we are moving toward a world where much more land is needed to meet the electricity supply demand. This occurs not only because the electric power needs are growing but also because the energy mix will comprise sources with inherently lower energy densities, which will cause the aggregated energy density of generation to reach half by 2050. A sixfold increase will occur in the spatial extent of power generation, from approximately 0.5% of land areas used for electric generation in 2020 to nearly 3.0% of land areas in 2050 (i.e., 430 million hectares of land). The world will be electrified by requiring an area roughly equal to the entire European Union (EU), which is one and a half times the size of India. The major contributor to increasing land use will be related to power generation from biomass, which is clearly seen in Fig. 15. Moreover, onshore wind power also drives up the spatial extent of electricity generation toward 2050. The estimated total of 3% of global land for electric generation in 2050 is significant. Even expanding cities are perceived to need much space. Nevertheless, they take up only 1% of global land^57^. Similarly, van Zalk and Behrens found a nearly fourfold increase in land use in the US from 2020 to 2050 based on the NREL 80% renewable energy scenario^3,58^.

When predicting land requirements, there are limitations to be mentioned. An additional challenge in the integration of more renewable sources is not only the land requirements but also the fact that power generation can be distributed across a larger area resulting in a higher need for infrastructure for electric power collection and further transmission. There is also an intermittency problem of renewable sources to meet the power adequacy requirements of the load. Residential users could act more flexibly, but there is an interest in industries relying on large amounts of electrical power to operate at a high capacity factor of consumption ($C_c$). Solutions to address this issue entail the introduction of a higher share of the ramping capability of the firm dispatchable power into the system^59^ or to realize large-scale energy storage^60^. Depending on the backup power solution or energy storage solution, these elements will occupy additional land and materials. Suppose the intermittency of renewables could be predicted more reliably^61^. In this case, this could reduce the amount of backup power needed to call on the demand. Recent research proposed ways to include the cost of storage in the cost of wind energy production^62^. Here, the spatial needs and additional infrastructure for these buffering services could also be included. In the case of storage such as hydrogen, the round-trip efficiency in a conservative estimate could reach as low as 35%^60^. In the extreme case, large portions of the power delivered by intermittent renewables must be fully buffered. This could generate rippling effects that could result in highly inefficient electricity generation. The equivalent annual energy density for this system could become even lower while the area required continued to increase. Finally, another layer on top as a result of increasing the share of clean electricity is that we would greatly expand the transmission capacity, at least triple the size toward 2050 according to IEA energy mix predictions.

**Table 19:** Predicted electricity generation based on the net-zero target toward 2050 (IEA NZE)^22^, assuming more than 20% of wind installations from 2021 to 2050 to be sea-based installations^22^, and 25% of the added wind energy generation (i.e., higher capacity factor).

Year	2020 (TWh)	2030 (TWh)	2040 (TWh)	2050 (TWh)
Solar PV	821	6970	17,031	23,469
Wind (onshore)	1501	6313	14,397	18,895
Hydro	4418	5870	7445	8461
Wind (offshore)	91	1695	4390	5890
Nuclear	2698	3777	4855	5497
Biomass	718	1407	2676	3279
Hydrogen-based turbines	0	875	1857	1713
Solar CSP	14	204	880	1386
Geothermal	94	330	625	821
Natural gas w/CCS	0	170	694	669
Coal w/CCS	4	289	966	663
Natural gas	6200	6222	626	253
Wave and tidal	2	27	77	132
Oil	756	189	6	6
Coal	9426	2947	0	0
Total generation ($E_g$)	26,778	37,316	56,553	71,164

**Table 20:** Projected land and sea use requirements under the NZE 2050 scenario in Table 19, where the required area ($A_g$) is estimated for individual power sources based on Eq. (6).

Year	2020	2030	2040	2050
Biomass$^{\text {a}}$	652,727 ${\text{km}^2}$	1,279,091 ${\text{km}^2}$	2,432,727 ${\text{km}^2}$	2,980,909 ${\text{km}^2}$
Wind (onshore)$^{\text {b}}$	79,000 ${\text{km}^2}$	332,263 ${\text{km}^2}$	757,737 ${\text{km}^2}$	994,474 ${\text{km}^2}$
Solar PV$^{\text {c}}$	9437 ${\text{km}^2}$	80,115 ${\text{km}^2}$	195,759 ${\text{km}^2}$	269,759 ${\text{km}^2}$
Wind (offshore)$^{\text {d}}$	2476 ${\text{km}^2}$	49,853 ${\text{km}^2}$	129,118 ${\text{km}^2}$	173,235 ${\text{km}^2}$
Hydro$^{\text {e}}$	14,926 ${\text{km}^2}$	19,831 ${\text{km}^2}$	25,152 ${\text{km}^2}$	28,584 ${\text{km}^2}$
Geothermal$^{\text {f}}$	2186 ${\text{km}^2}$	7674 ${\text{km}^2}$	14,535 ${\text{km}^2}$	19,093 ${\text{km}^2}$
Solar CSP$^{\text {g}}$	79 ${\text{km}^2}$	1146 ${\text{km}^2}$	4944 ${\text{km}^2}$	7787 ${\text{km}^2}$
Wave and tidal$^{\text {h}}$	42 ${\text{km}^2}$	568 ${\text{km}^2}$	1621 ${\text{km}^2}$	2779 ${\text{km}^2}$
Hydrogen-based turbines$^{\text {i}}$	0 ${\text{km}^2}$	1040 ${\text{km}^2}$	2208 ${\text{km}^2}$	2037 ${\text{km}^2}$
Coal w/CCS$^{\text {j}}$	5 ${\text{km}^2}$	365 ${\text{km}^2}$	1219 ${\text{km}^2}$	836 ${\text{km}^2}$
Nuclear$^{\text {k}}$	403 ${\text{km}^2}$	563 ${\text{km}^2}$	724 ${\text{km}^2}$	820 ${\text{km}^2}$
Natural gas w/CCS$^{\text {l}}$	0 ${\text{km}^2}$	67 ${\text{km}^2}$	275 ${\text{km}^2}$	265 ${\text{km}^2}$
Natural gas$^{\text {m}}$	1890 ${\text{km}^2}$	1896 ${\text{km}^2}$	191 ${\text{km}^2}$	77 ${\text{km}^2}$
Oil$^{\text {n}}$	480 ${\text{km}^2}$	120 ${\text{km}^2}$	4 ${\text{km}^2}$	4 ${\text{km}^2}$
Coal$^{\text {o}}$	8494 ${\text{km}^2}$	2656 ${\text{km}^2}$	0 ${\text{km}^2}$	0 ${\text{km}^2}$
Total land use	769,627 ${\text{km}^2}$	1,726,827 ${\text{km}^2}$	3,435,475 ${\text{km}^2}$	4,304,645 ${\text{km}^2}$
Total land and sea use ($A_g$)	772,145 ${\text{km}^2}$	1,777,248 ${\text{km}^2}$	3,566,214 ${\text{km}^2}$	4,480,659 ${\text{km}^2}$
Total generation ($E_g$)	26,778 TWh	37,316 TWh	56,553 TWh	71,164 TWh
Generation density ($\varepsilon _g$)	34.680 ${{\text { GWh/km}}^2}$	20.997 ${{\text { GWh/km}}^2}$	15.858 ${{\text { GWh/km}}^2}$	15.882 ${{\text { GWh/km}}^2}$
Consumption density ($\varepsilon _c$)	0.180 ${{\text { GWh/km}}^2}$	0.251 ${{\text { GWh/km}}^2}$	0.380 ${{\text { GWh/km}}^2}$	0.478 ${{\text { GWh/km}}^2}$
Normalized spatial area ($\varepsilon _c/\varepsilon _g$)	0.519%	1.195%	2.396%	3.010%

This is based on a mean energy density assumption, with values retrieved from the data summarized in Table 16. The remaining values are obtained from other sources specified in the footnote.

$^{\text {a}}$Biomass^3^: 0.0011 ${{\text {TWh/km}}^2}$. $^{\text {b}}$Wind (onshore): 0.019 ${{\text {TWh/km}}^2}$. $^{\text {c}}$Solar PV: 0.087 ${{\text {TWh/km}}^2}$.

$^{\text {d}}$Wind (offshore): 0.034 ${{\text {TWh/km}}^2}$. $^{\text {e}}$Hydro: 0.296 ${{\text {TWh/km}}^2}$. $^{\text {f}}$Geothermal: 0.043 ${{\text {TWh/km}}^2}$.

$^{\text {g}}$Solar CSP: 0.178 ${{\text {TWh/km}}^2}$. $^{\text {h}}$Wave and tidal: 0.0475 ${{\text {TWh/km}}^2}$ (assuming equal shares of wave and tidal power).

$^{\text {i}}$Hydrogen-based turbines^63^: 0.841 ${{\text {TWh/km}}^2}$ (assuming 3 times more space than that needed for natural gas w/CCS by retrofitting pipelines). The NZE predicts 60% green fuel from electrolysis and 40% blue fuel from natural gas^22^.

$^{\text {j}}$Coal w/CCS^3^: 0.793 ${{\text {TWh/km}}^2}$ (40% more land use than that w/o CCS^26^). $^{\text {k}}$Nuclear: 6.703 ${{\text {TWh/km}}^2}$.

$^{\text {l}}$Natural gas w/CCS: 2.523 ${{\text {TWh/km}}^2}$ (30% more land use than that w/o CCS^26^). $^{\text {m}}$Natural gas: 3.280 ${{\text {TWh/km}}^2}$.

$$^{\text {n}}$$Oil^3^: 1.573 $${{\text {TWh/km}}^2}$$. $$^{\text {o}}$$Coal^3^: 1.110 $${{\text {TWh/km}}^2}$$.

## Conclusion

This paper revealed that the land and sea requirements for future power generation facilities are currently projected to significantly change by 2050. The obtained annual energy densities for 870 real-world power sources were used to estimate the environmental footprint of the future energy mix. A sixfold increase in the spatial extent of the worldwide power generation resulted not only result from the fact that new renewable energies are more challenging to harvest than the existing mix of sources but also from the fact that global electrification will experience a threefold increase by 2050.

Our paper provided evidence that, in a worldwide sense, hydropower is the most energy-dense renewable source. However, this is not the case when one considers certain regions, e.g., where the topography does not favor hydropower generation or in areas where the performance of solar power is much higher than the global average. It must also be emphasized that hydropower exhibited the highest standard deviation among the investigated sources. The standard deviation of the annual energy density ranged from 0 to 1.67 ${{\text {TWh/km}}^2}$. The upper standard deviation of hydropower was very close to the lower standard deviation of nuclear power, at 1.88 ${{\text {TWh/km}}^2}$, but far higher than that of the natural gas population.

Contrary to conventional wisdom, our work also demonstrated that nuclear power exhibits a higher annual generation density than that of natural gas power plants, considering the land occupation of pipelines and mining to feed gas-fired power plants. In this paper, the generation density of a nuclear power plant included the safety surface in addition to the nuclear power plant itself.

While biomass is by far the most dilute renewable energy source, this paper found through a population study of 148 specimens that onshore wind farms are the second most dilute source for power generation based on the assumptions of the spatial extent outlined in this paper. Even though our paper confirmed the order of magnitude in earlier studies, the limitations of the assumptions should be stated. Our calculations considered the total site area where wind farms are distributed. There occur empty areas between wind turbines that could be utilized for grazing, agriculture, and recreation. When the occupied area of wind power only considers tower footprints and access roads, the specific power could easily increase by at least an order of magnitude^26^. However, this does not fully represent the high spacing between the distributed sources of a wind farm and the low scalability in space-limited regions. High land requirements also generate significant implications for the need for materials and infrastructure to collect energy from wind turbines. There exist potential indirect effects on wildlife and degradation of the quality of landscapes^64^, and the visual footprint is significant throughout the entire area. The wind power performance could be enhanced in the future via technological improvements such as wake steering, which could be used to enhance the annual energy production of spaced turbines in wind farms^65^.

## Supplementary Information

Supplementary Information 1.

Supplementary Information 2.

Supplementary Information 3.

Supplementary Information 4.

Supplementary Information 5.

Supplementary Information 6.

Supplementary Information 7.

Supplementary Information 8.

## Data Availability

All collected data in this study are included in the published article (and Supplementary Information files).

## List of symbols

$\eta _g$: Electrical energy conversion efficiency of generation facility, [%] or [pu]
$\varepsilon _g$, $\varepsilon _c$: Annual energy density (spatially) of the generation and consumption, $[{\text{TWh/km}}^{2}]$, $[{\text{GWh/km}}^{2}]$, or $[{\text{J/m}}^2]$
$A_g$, $A_s$: Required area of the generation facility and energy source, [${\text{km}^{2}}$] or $[{\text{m}^{2}}]$
$A_{min}$, $A_{max}$, $A_{tot}$: Minimum and maximum available areas for power generation and the total area, $[{\text{km}^{2}}]$ or $[{\text{m}^{2}}]$
*avg*, *dev*: Mean and deviation values of the dataset
$C_g$, $C_c$: Capacity factor or load factor of power generation and consumption, [%] or [pu]
$E_g$, $E_c$: Electrical energy generated and consumed, [TWh] or [J]
*mdn*, *max*: Median and maximum values of the dataset
*n*: Number of samples in the population
$p_g$, $p_s$, $p_c$: Mean power density (spatially) of the generation facility, energy source, and consumption, [${\text{W/m}}^{2}$]
$P_s$, $P_g$, $P_c$: Power of the source and rated electrical power of generation and consumption, [MW] or [W]
$T_y$: Total time of the entire year, $\approx$ 8765.8 h
